# Antimicrobial susceptibility, multilocus sequence typing, and virulence of *listeria* isolated from a slaughterhouse in Jiangsu, China

**DOI:** 10.1186/s12866-021-02335-7

**Published:** 2021-11-25

**Authors:** Liting Wu, Hongduo Bao, Zhengquan Yang, Tao He, Yuan Tian, Yan Zhou, Maoda Pang, Ran Wang, Hui Zhang

**Affiliations:** 1 Jiangsu Key Laboratory for Food Quality and Safety-State Key Laboratory Cultivation Base of MOST, Institute of Food Safety and Nutrition, Jiangsu Academy of Agricultural Sciences, No 50 Zhongling Street, Nanjing, 210014 Jiangsu China; 2 College of Food Science and Engineering, Yangzhou University, Yangzhou, 225009 China; 3 Jiangsu University – School of Food and Biological Engineering, Zhenjiang, 212013 China

**Keywords:** Antimicrobial resistance, Antimicrobial resistance genes, *Listeria*, MLST, Virulence

## Abstract

**Background:**

*Listeria monocytogenes* is one of the deadliest foodborne pathogens. The bacterium can tolerate severe environments through biofilm formation and antimicrobial resistance. This study aimed to investigate the antimicrobial susceptibility, resistance genes, virulence, and molecular epidemiology about *Listeria* from meat processing environments.

**Methods:**

This study evaluated the antibiotic resistance and virulence of *Listeria* isolates from slaughtering and processing plants. All isolates were subjected to antimicrobial susceptibility testing using a standard microbroth dilution method. The harboring of resistant genes was identified by polymerase chain reaction. The multilocus sequence typing was used to determine the subtyping of the isolates and characterize possible routes of contamination from meat processing environments. The virulence of different STs of *L. monocytogenes* isolates was evaluated using a Caco-2 cell invasion assay.

**Results:**

A total of 59 *Listeria* isolates were identified from 320 samples, including 37 *L. monocytogenes* isolates (62.71%). This study evaluated the virulence of *L. monocytogenes* and the antibiotic resistance of *Listeria* isolates from slaughtering and processing plants. The susceptibility of these 59 isolates against 8 antibiotics was analyzed, and the resistance levels to ceftazidime, ciprofloxacin, and lincomycin were as high as 98.31% *(L. m* 37; *L. innocua* 7; *L. welshimeri* 14), 96.61% (*L. m* 36; *L. innocua* 7; *L. welshimeri* 14), and 93.22% (*L. m* 35; *L. innocua* 7; *L. welshimeri* 13), respectively. More than 90% of the isolates were resistant to three to six antibiotics, indicating that *Listeria* isolated from meat processing environments had high antimicrobial resistance. Up to 60% of the isolates harbored the tetracycline-resistance genes *tetA* and *tetM*. The frequency of *ermA*, *ermB*, *ermC*, and *aac(6′)-Ib* was 16.95, 13.56, 15.25, and 6.78%, respectively. Notably, the resistant phenotype and genotype did not match exactly, suggesting that the mechanisms of antibiotic resistance of these isolates were likely related to the processing environment. Multilocus sequence typing (MLST) revealed that 59 *Listeria* isolates were grouped into 10 sequence types (STs). The dominant *L. monocytogenes* STs were ST5, ST9, and ST121 in the slaughtering and processing plant of Jiangsu province. Moreover, ST5 subtypes exhibited high invasion in Caco-2 cells compared with ST9 and ST121 cells.

**Conclusion:**

The dominant *L. monocytogenes* ST5 persisted in the slaughtering and processing plant and had high antimicrobial resistance and invasion characteristics, illustrating a potential risk in food safety and human health.

## Introduction

*Listeria monocytogenes* is one of the most important foodborne pathogens. The bacterium can infect humans and animals and cause meningoencephalitis, abortion, and sepsis, resulting in high rates of infection and mortality [1]. *L. monocytogenes* can persist in food processing environments, such as meat, poultry, dairy, and seafood processing facilities, and the bacteria can proliferate during the storage of chilled food products due to its high degree of resistance under harsh conditions [1]. Sources and contamination patterns in various types of products have not yet been determined. The occurrence of *L. monocytogenes* in the food processing environment is variable, whereas, in food, it is generally around 5%–6% [2]. The food processing environment is easily contaminated by *L. monocytogenes* [3]. The molecular typing of isolates, including pulsed-field gel electrophoresis (PFGE) inside and outside a food processing facility, can indicate potential sources of contamination from the external environment [4]. With the development of whole-genome sequencing (WGS), multilocus sequence typing (MLST) has been widely used for the epidemiological investigation of *L. monocytogenes* and source tracking of specific strains during outbreaks. Thus, the main ST subtypes can be analyzed more accurately [5]. Antimicrobial resistance is a global public health problem [6–8]. *L. monocytogenes* rarely develops acquired resistance to antibiotics. However, researchers have reported that *L. monocytogenes* is resistant to antibiotics such as tetracycline, ciprofloxacin, erythromycin, and ampicillin [1]. Several recent studies have reported an increased rate of resistance to one or more clinically relevant antibiotics in environmental isolates [7, 9, 10] and less frequently in clinical strains [11, 12]. The prevalence of antimicrobial resistance has increased due to the seriousness of multidrug resistance and the transmission of resistance genes between bacteria and across species.

However, precise information on the ancestral and evolutionary linkage and the genetic diversity of *L. monocytogenes* is presently not available*.* The advent of subtyping techniques, such as PFGE and WGS, has enabled source tracking of *L. monocytogenes* during outbreak investigations. However, these technologies are not yet used for general surveillance in food supply chains due to their cost, complexity of analysis, and expertise required to interpret such data. In the present study, we used MLST, which could determine the source of processing environment contamination by analyzing slaughtering operations, to trace the presence of *L. monocytogenes* in isolates from food commodities. The method allowed us to perform subtyping of the pathogen and characterize possible routes of contamination.

## Results

### Occurrence of *listeria* spp. in the processing environment

The overall prevalence of *Listeria* in slaughter and processing environments tested in 2019 is shown in Table 1. Thirty-seven isolates of *L. monocytogenes*, 7 isolates of *L. innocua,* and 15 isolates of *L. welshimeri* were obtained. The 59 isolates were distributed in different areas: 7 from the slaughter area (8.75), 9 from the cutting and deboning room (11.25%), 23 from the visceral area (28.75%), and 20 from the meat cooling and refrigeration area (25.00%). A total of 59 *Listeria* isolates were recovered from 320 analyzed samples (18.44%), including 37 *L. monocytogenes* (11.56%). The highest percentage of *L. monocytogenes* strains (13) was found in samples taken from the cooling and refrigeration area (Table 1). Moreover, group 1/2b was the main serotype (12/37, Table 2), and the next highest were 1/2a (7/37) and 1/2c (7/37). The others were 3a (3/37), 3b (5/37), and 3c (3/37). Hence, serotypes 1/2b, 1/2a, and 3b were the main endemic *L. monocytogenes* isolates in slaughtering environments.

**Table 1:** Isolation frequency of *Listeria* from pig slaughter factory

Sample type	No. of samples	No. of *Listeria*	No. of positive samples (%)
Slaughter area (A)	80	*L. monocytogenes* (6) *L. innocua* (1)	7 (8.75)
Cutting and deboning room (B)	80	*L. monocytogenes* (7) *L. innocua* (2)	9 (11.25)
Visceral area (C)	80	*L. monocytogenes* (11) *L. innocua* (1) *L. welshimeri* (11)	23 (28.75)
Meat cooling and refrigeration area (D)	80	*L. monocytogenes* (13) *L. innocua* (3) *L. welshimeri* (4)	20 (25.00)
Total	320	*L. monocytogenes* (37) *L. innocua* (7) *L. welshimeri* (15)	59 (18.44)

Phylogenetic groups of tested *L. isteria* strains (*n* = 59)

**Table 2:** Serotypes and isolation regions of *L. monocytogenes* isolates

Group	Number of *L. monocytogenes* isolates [n(%)]				Total
	Slaughter area (A)	Cutting and deboning room (B)	Visceral area (C)	Meat cooling and refrigeration area (D)	
1/2a	ND	ND	LM3–11	LM1, LM2, LM3, LM6, LM7, LM8,	7 (18.91%)
1/2b	LMA1, LMA8, LMA9, LMA13, LMA-II	LMB4, LMB-I	LMC4, LMC9, LMC15, LMC-I	LMD3, LMD10	13 (35.14%)
1/2c	ND	LM2–18	LM3–2-2,LM3–19, LM3–20-2	LM1T7, LM2T3, LM2W3	7 (18.92%)
3a	LM1–9	ND	LMC11	LM4	3 (8.11%)
3c	ND	ND	LMX-3, LMC7	LM1W3	3 (8.11%)
3b	ND	LMB5, LMB9, LMB10, LMB13	ND	ND	4 (10.81%)
Total	6	7	11	13	37 (100%)

ND represents “None determined

### Antimicrobial susceptibility testing

**Fig. 1:**
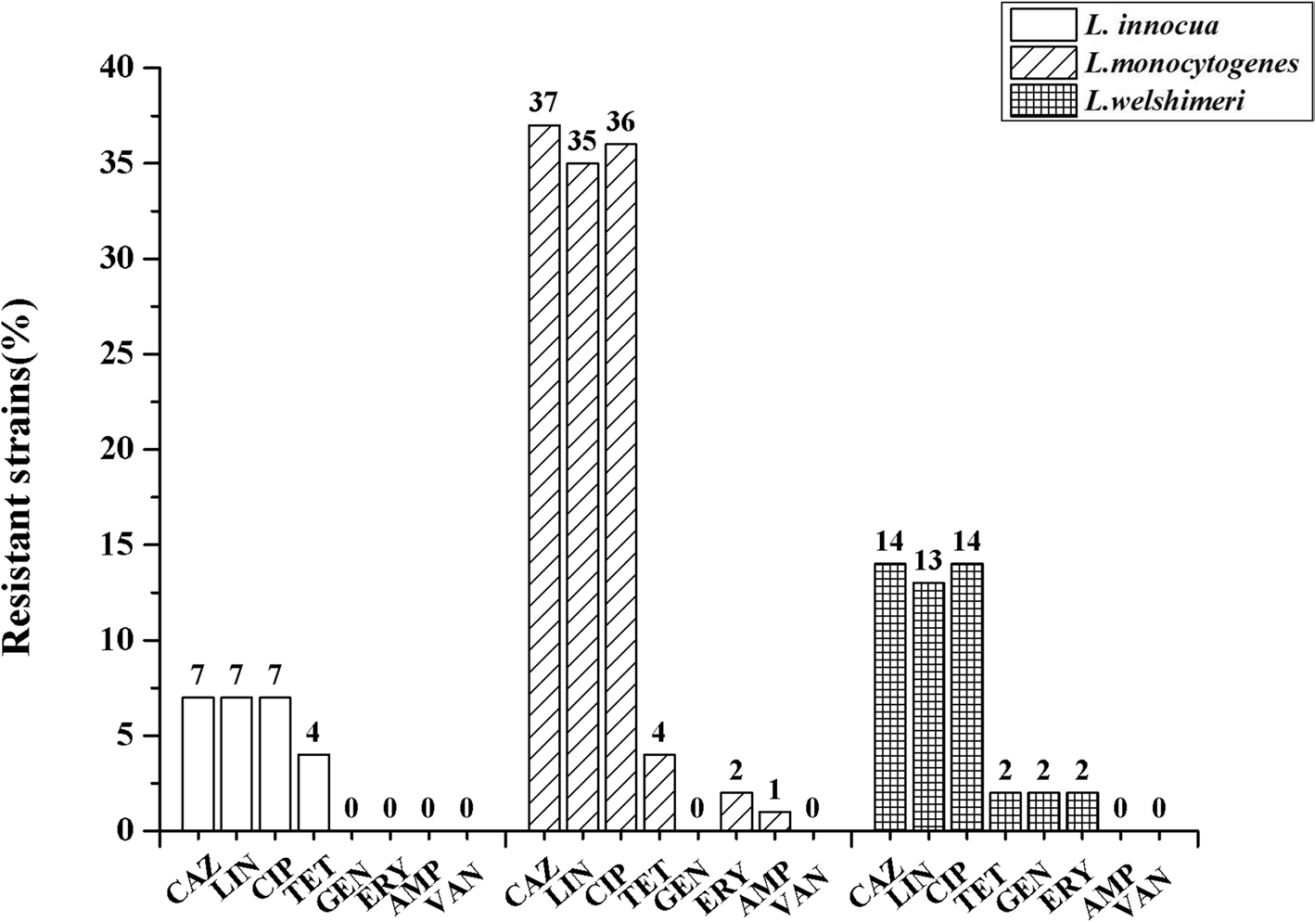
Resistant analysis of *L. welshimeri*, *L. inocua* and *L. monocytogenes*. Gentamicin (GEN), ampicillin (AMP), ceftazidime (CAZ), ciprofloxacin (CIP), tetracycline (TET), erythromycin (ERY), lincomycin (LIN) and vancomycin (VAN) were selected as test antibiotics. *Streptococcus pneumonia* ATCC 49619 was selected as the quality control strain

**Fig. 2:**
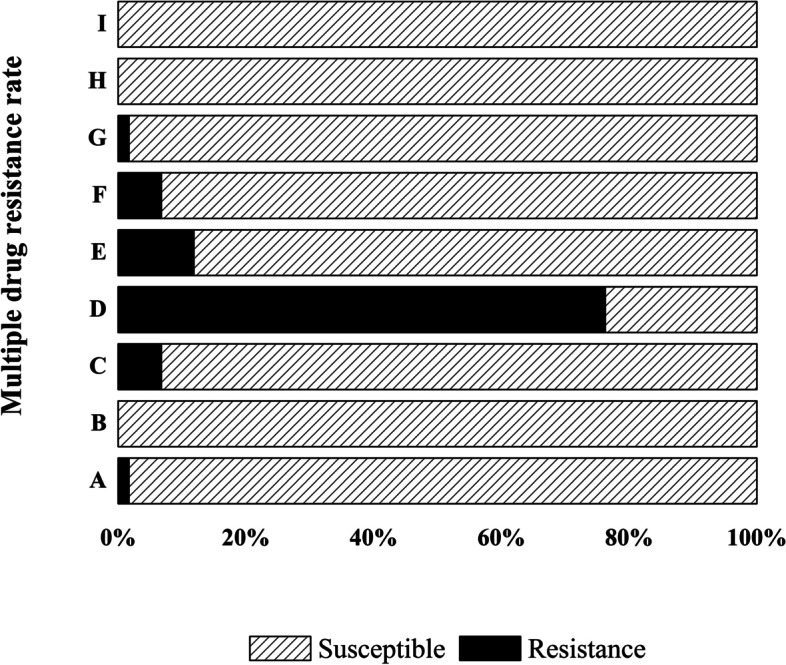
Antimicrobial susceptibility of *L. monocytogenes* isolates from processing plants to eight antibiotics. Gentamicin (GEN), ampicillin (AMP), ceftazidime (CAZ), ciprofloxacin (CIP), tetracycline (TET), erythromycin (ERY), lincomycin (LIN) and vancomycin (VAN) were selected as test antibiotics. A:non-resistance, B:one-resistance, C:two-resistance, D:three-resistance, E:four-resistance, F: five-resistance, G:six-resistance, H:seven-resistance, I:eight-resistance. *Streptococcus pneumonia* ATCC 49619 was selected as the quality control strain

The susceptibility of the 59 isolates to 8 antibiotics was examined using the microbroth dilution method. The results showed that the isolates were resistant to ceftazidime (MIC ≥32 μg/mL; 58/59, 98.31%), ciprofloxacin (MIC ≥64 μg/mL; 57/59, 96.61%), and lincomycin (MIC ≥4 μg/mL; 55/59, 93.22%). The resistance to tetracycline reached 16.95% (MIC ≥16 μg/mL; 10/59). Very few isolates were resistant to gentamicin (MIC 16 μg/mL; 2/59) or ampicillin (MIC 32 μg/mL; 1/59). Noteworthy was the intermediate resistance against erythromycin (MIC = 1–4 μg/mL; 29/59) observed in these isolates. All of the isolates were highly susceptible to vancomycin (100%) (Table 3). It was obvious that *L. monocytogenes*, *L. innocua*, and *L. welshimeri* were mainly resistant to ceftazidime, lincomycin, and ciprofloxacin. Multidrug resistance showed that 58 strains were resistant to at least 2 antibiotics (Fig. 1). The proportion of the strains resistant to three kinds of antibiotics was 76.27%, and the proportion of the strains resistant to three to six antibiotics was 91.38% (Fig. 2). Only one isolate (LM3–2) of *L. welshimeri* was susceptible to all antibiotics. The *L. welshimeri* isolate LM3–7 from the slaughter area was resistant to six antibiotics, and the resistance was the most serious in this study (Figs. 1, 2, and 4).

**Table 3:** Antimicrobial-resistance profiles of *Listeria* isolates from the four areas (n = 59)

Source and no. of resistant strains (%)						
Antibitocs (ug/ml)		Slaughter area *n* = 7	Cutting and deboning room *n* = 9	Visceral area *n* = 23	Meat cooling and refrigeration area *n* = 20	Total
GEN	R ≥ 16	0 (0.00%)	0 (0.00%)	*L. welshimeri* (1) (4.35%)	*L. welshimeri* (1) (5.00%)	2 (3.39%)
	I = 8	0 (0.00%)	0 (0.00%)	*L.m* (1) (4.35%)	0 (0.00%)	1 (1.69%)
	S ≤ 4	*L. innocua* (1) *L. m*(6) (100.00%)	*L. innocua* (2) *L. m* (7) (100.00%)	*L. innocua* (1) *L. welshimeri* (9) *L. m* (11) (91.30%)	*L. innocua* (3) *L. welshimeri* (3) *L. m*(13) (95.00%)	56 (94.92%)
CAZ	R ≥ 32	*L. innocua* (1) *L. m*(6) (100.00%)	*L. innocua* (2) *L. m* (7) (100.00%)	*L. innocua* (1) *L. welshimeri*(10) *L. m* (11) (95.65%)	*L. innocua* (3) *L. welshimeri* (4) *L. m* (13) (100.00%)	58 (98.31%)
	I = 16	0 (0.00%)	0 (0.00%)	0 (0.00%)	0 (0.00%)	0 (0.00%)
	S ≤ 8	0 (0.00%)	0 (0.00%)	*L. welshimeri* (1) (4.35%)	0 (0.00%)	1 (1.70%)
AMP	R ≥ 32	*L. m* (1) (14.29%)	0 (0.00%)	0 (0.00%)	0 (0.00%)	1 (1.69%)
	I = 16	0 (0.00%)	0 (0.00%)	0 (0.00%)	0 (0.00%)	0 (0.00%)
	S ≤ 8	*L. innocua* (1) *L. m* (5) (85.71%)	*L.innocua* (2) *L. m* (7) (100.00%)	*L. innocua* (1) *L. welshimeri* (11) *L. m* (11) (100.00%)	*L. innocua* (3) *L. welshimeri* (4) *L. m* (13) (100.00%)	58 (98.31%)
CIP	R ≥ 4	*L. innocua* (1) *L. m* (6) (100.00%)	*L. innocua* (2) *L. m* (6) (88.89%)	*L. innocua* (1) *L. welshimeri*(10) *L. m* (11) (95.65%)	*L. innocua* (3) *L. welshimeri* (4) *L. m* (13) (100.00%)	57 (96.61%)
	I = 2	0 (0.00%)	0 (0.0%)	*L. welshimeri* (1) (4.35%)	0 (0.0%)	1 (1.69%)
	S ≤ 1	0 (0.00%)	*L. m* (1) (11.11%)	0 (0.00%)	0 (0.00%)	1 (1.69%)
TET	R ≥ 16	*L. m* (1) (14.29%)	*L. innocua* (2) *L. m* (1) (33.33%)	*L. innocua* (1) *L. welshimeri* (2) *L. m* (1) (17.39%)	*L. innocua* (1) *L. m* (1) (10.00%)	10 (16.95%)
	I = 8	0 (0.00%)	0 (0.00%)	0 (0.00%)	0 (0.00%)	0 (0.00%)
	S ≤ 4	*L. innocua* (1) *L. m* (5) (85.71%)	*L. m* (6) (72.7%)	*L. welshimeri* (9) *L. m* (10) (82.61%)	*L. innocua* (2) *L. welshimeri* (4) *L. m* (12) (90.00%)	49 (83.05%)
ERY	R ≥ 8	0 (0.00%)	0 (0.00%)	*L. welshimeri* (1) *L. m* (1) (8.70%)	*L. welshimeri* (1) *L. m* (1) (10.00%)	4 (6.78%)
	I = 1–4	*L. m* (2) (28.57%)	*L. innocua* (2) *L. m* (4) (66.67%)	*L. innocua* (1) *L. welshimeri* (7) *L. m* (3) (47.83%)	*L. welshimeri* (2) *L. m* (8) (50.00%)	29 (49.15%)
	S ≤ 0.5	*L. innocua* (1) *L. m* (4) (71.43%)	*L. m* (3) (33.33%)	*L. welshimeri*(3) *L. m* (7) (43.48%)	*L. innocua* (3) *L. welshimeri*(1) *L. m* (4) (40.00%)	26 (44.07%)
LIN	R ≥ 4	*L. innocua* (1) *L. m* (5) (85.71%)	*L. innocua* (2) *L. m* (7) (100.00%)	*L. innocua* (1) *L. welshimeri* (9) *L. m* (11) (91.30%)	*L. innocua* (3) *L. welshimeri* (4) *L. m* (12) (95.00%)	55 (93.22%)
	I = 1–2	*L. m* (1) (14.29%)	0 (0.00%)	*L. welshimeri* (2) (8.70%)	*L. m* (1) (5.00%)	4 (6.78%)
	S -	0 (0.00%)	0 (0.00%)	0 (0.00%)	0 (0.00%)	0 (0.00%)
VAN	R ≥ 32	0 (0.00%)	0 (0.00%)	0 (0.00%)	0 (0.00%)	0 (0.00%)
	I = 8–16	0 (0.00%)	0 (0.00%)	0 (0.00%)	0 (0.00%)	0 (0.00%)
	S ≤ 4	*L. innocua* (1) *L. m* (6) (100.00%)	*L. innocua* (2) *L. m* (7) (100.00%)	*L. innocua* (1) *L. welshimeri* (11) *L. m* (11) (100.00%)	*L. innocua* (3) *L. welshimeri* (4) *L. m* (13) (100.00%)	59 (100.00%)

*GEN* gentamicin, *CAZ* ceftazidime, *AMP* ampicillin, *CIP* ciprofloxacin, *TET* tetracycline, *ERY* erythromycin, *LIN* lincomycin, *VAN* vancomycin

**Fig. 3:**
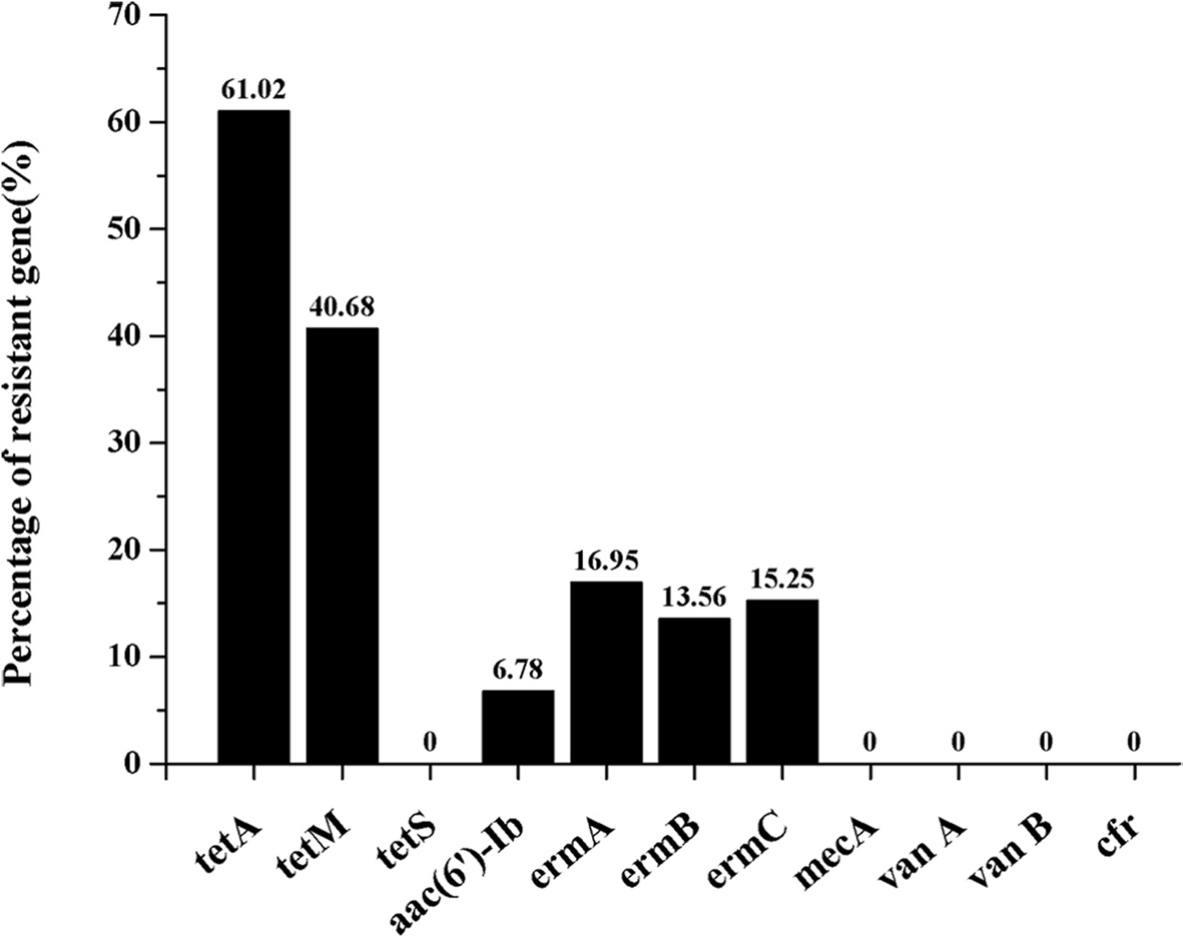
Resistance genotypes of 59 *Listeria* isolates. Eleven resistance genes *tetA*, *tetM*, *tetS*, *ermA*, *ermB*, *ermC*, *aac*(6′)*-Ib*, *mecA*, *vanA*, *vanB,* and *cfr* were selected as specific resistance genes and were identified by PCR within *Listeria* spp. . Primers used in this study are listed in Table 5

The prevalence of 11 resistance genes was assessed; the results are summarized in Table 4. In the slaughtering and processing environment, the genes *tetA*, *tetM*, *ermA*, *ermB*, *ermC*, and *aac*(*6′*)*-Ib* were detected in different areas. The *tetS*, *mecA*, vanA, *vanB*, and *cfr* genes were not detected in all *Listeria* isolates. Tetracycline-resistant genes *tetA* (61.3%) and *tetM* (45.3%) were the two most commonly detected antibiotic-resistant genes. The erythromycin-resistant gene cassette, including *ermA* (16.95%), *ermB* (13.56%), and *ermC* (15.25%), was present among *L. monocytogenes*, *L. welshimeri*, and *L. innocua*. Four isolates of *Listeria* were found to carry *aac*(*6′*)*-Ib* by detecting the resistance gene for aminoglycosides. However, the resistant genotypes and phenotypes were not exactly the same (Table 4 and Fig. 3). In comparison, 58 strains of ceftazid-resistant isolates were found, but none of these isolates presented known resistance genes (Table 3).

**Table 4:** Correlation rate of phenotype and genotype of the *Listeria spp*

Antibiotics	Resistant strains	Resistance genes	Resistance genes strains
Tetracycline	9	*tetA*	36
		*tetM*	24
		*tetS*	0
Ciprofloxacin	57	*aac(6′)-Ib*	4
Eryphilin	4	*ermA*	10
		*ermB*	8
		*ermC*	9
Ceftazidime	58	*mecA*	0
Vancomycin	0	*van A*	0
		*van B*	0

### MLST

**Fig. 4:**
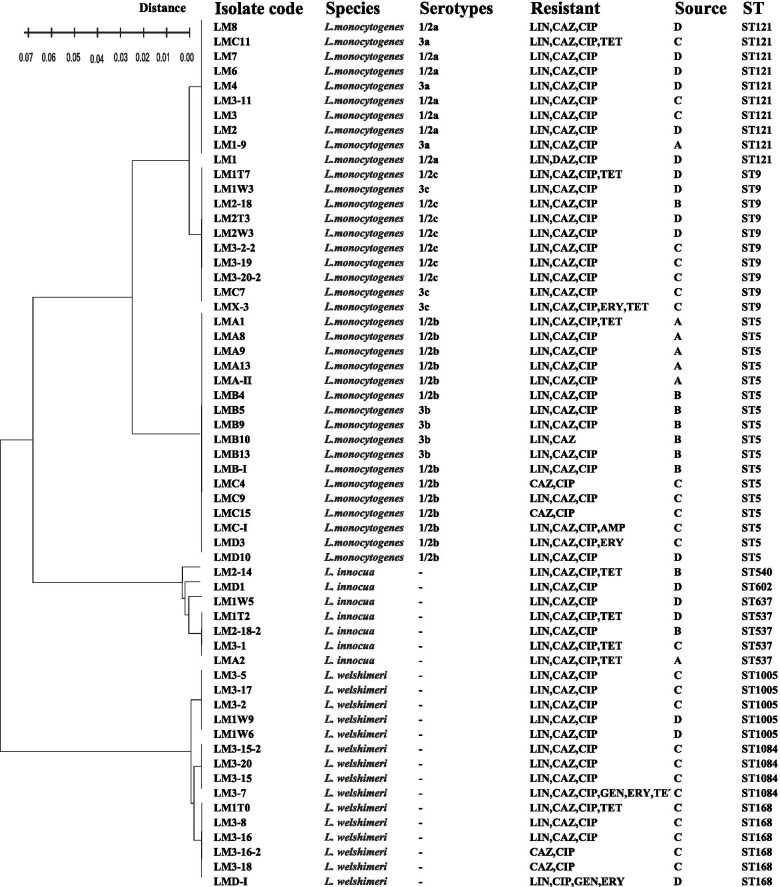
Serotypes, resistance, source, and STs of the *Listeria* isolates from the processing environment. MLST performed based on seven housekeeping genes (*abcZ, bglA, cat, dapE, dat, ldh and lhkA*) according to the previous method. Genotypic data are available at http://bigsdb.web.pasteur.fr/listeria/. Minimum spanning tree analysis was inferred using BioNumerics (Version 5.10, Applied Maths, Belgium). ND represents: None determined

A total of 59 *Listeria* isolates were classified into 10 sequence types (STs) (Fig. 4). Seventeen *L. monocytogenes* belonged to ST5 (17/37, 45.95%). Other STs belonged to ST9 (10/37) and ST121 (10/37). The remaining 22 non-*L. monocytogenes* isolates were grouped into ST540, ST602, ST637, ST537, ST10057, ST168, and ST1084. The most endemic ST was ST5, which was isolated from four areas. ST121 was widely distributed in the meat cooling and refrigeration area (D). Seven *L. innocua* isolates were divided into four STs, four of which belonged to ST537. Fifteen *L. welshimeri* isolates were divided into ST1005, ST1084, and ST168. The 13 isolates of *L. monocytogenes* ST5 belonged to the serotype 1/2b, while 4 belonged to the serotype 3b. Among the 10 ST9 isolates, 3 belonged to the serotype 3c, while 7 belonged to the serotype 1/2c. The serotypes of ST121 consisted of seven isolates of 1/2a and three isolates of 3a. We found that isolates classified as the same serogroup could be differentiated into different STs. This finding may be applied to other isolates of *L. monocytogenes.*

### Virulence genes and invasion assays

**Fig. 5:**
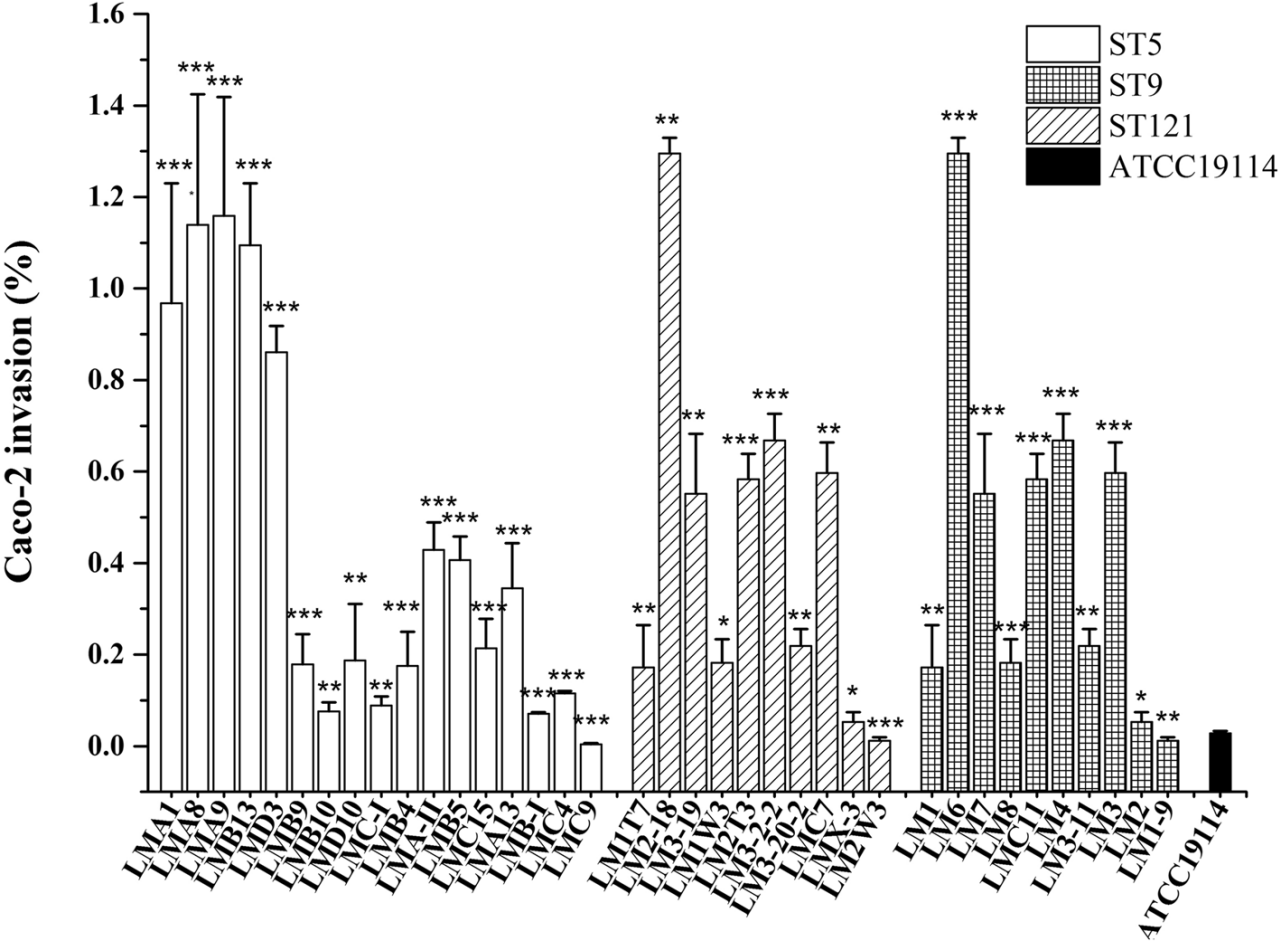
Invasion level of *L. monocytogenes* isolates against the human colorectal adenocarcinoma cell line Caco-2 cells. In vitro invasion was performed in the Caco-2 cell line (3.0 × 10^5^ cells per well) infected with 1.0 × 10^7^ - 2.0 × 10^7^ *L. monocytogenes* cells/well. After contact for 90 min, viable intracellular bacteria were enumerated by plating appropriate dilutions of the cell lysate on BHI agar. Error bars represent standard deviations of the mean. ATCC19114 strain was included as an invasion control. Significant difference compared with ATCC19114; *** *P* < 0.01; ** *P* < 0.05; *P > 0.05

In this study, the virulence and invasiveness of *L. monocytogenes* were evaluated using invasion assays. Seven virulence-associated genes (*prfA*, *plcA*, *gyrB*, *plcB*, *inlA*, *hly*, and *sigB*) were detected by polymerase chain reaction (PCR). Each of the seven virulence-associated genes was detected in all *L. monocytogenes* strains. The invasion efficiency of the isolates ranged from 0.002 to 1.295%. The results showed that the isolates within the same STs had different levels of invasiveness against Caco-2 cells. The invasion frequencies of ST5 and ST121 ranged from 0.004 to 1.159% and 0.012 to 1.295%, respectively. The invasion frequency of ST9 was relatively lower than that of ST5 and ST121, from 0.002 to 0.669%. The average invasion frequency of ST9 was 0.1406%, whereas the values for ST5 and ST121 were 0.4419 and 0.4332%, respectively (Fig. 5). The ST5 isolates mainly came from the cutting and deboning room and visceral area regions and showed higher levels of invasiveness.

## Discussion

*L. monocytogenes*, which is ubiquitous in the environment, is the causative agent of listeriosis. The incidence of the disease is low compared with the incidence of diseases caused by other foodborne pathogens; however, the disease outcome is often more serious [13]. Food safety regulations in many countries have tended to adopt a zero-tolerance policy for *L. monocytogenes* in ready-to-eat (RTE) food products, as human listeriosis outbreaks have been most often associated with RTE products consumed without prior cooking. RTE meat products contaminated with *Listeria* might be the result of cross-contamination during processing and handling during storing, slicing, weighing, and packaging [14]. In this study, we investigated the resistance and STs of the *Listeria* isolated from the slaughter and processing environment in Jiangsu province, China. Fifty-nine *Listeria* strains were found in 320 samples from the slaughterhouse (18.44%), including 37 of *L. monocytogenes* (37), 7 of *L. innocua* (7), and 15 of *L. welshimeri* (15). Antunes et al. [15] found that *Listeria spp.* were present in all 63 (100%) poultry samples, including *L. innocua* (32 isolates) and *L. welshimeri* (8 isolates). Yadav et al. [16] reported that the 20 strains of *L. innocua* were isolated from 2417 animals and their surrounding environment samples. Thus, it was inferred that the pattern of susceptibility between *L. monocytogenes* and *L. innocua* was important, because both species were usually found in the same food or food processing environment [7]. However, previous reports showed that 19 cheese factories (55.8%) were contaminated with *Listeria* spp., demonstrating a higher contamination rate compared with that reported in our study. Of these, 20.6% were *L. monocytogenes* positive, while in our study, the proportion reached 62.71%. Moreover, *L. monocytogenes* was found on 4.9% of product contact surfaces and 18.8% of floor drains [17]. In Romania, meat processing plants were contaminated at higher prevalence rates of *L. monocytogenes* (18.8 and 26.5%) [18–20]. Vongkamjan et al. [21] also demonstrated that *L. monocytogenes* was found (35%) in environmental samples from one seafood processing plant. Therefore, the environmental surfaces appear to be easier to contaminate than the food matrices [22]. In our study, the prevalence of *L. monocytogenes* in meat cooling and refrigeration areas (D) was significantly higher than that in other areas in 2018. The suggestion that *L. monocytogenes* grows well at low temperatures should be remembered. Therefore, periodic surveillance and sanitation should be strictly implemented to improve the hygiene conditions of the slaughter and processing environment and hence achieve higher food safety levels.

Previous studies reported that *L. monocytogenes* from seafood processing plants belonged to serotypes 1/2b, 3b, 4b, 4d, and 4e [17, 23, 24], and serotypes 1/2b, 1/2a, 4b, and 1/2c were usually found in meat products and meat processing plants [25]. Skowron et al. found that most (38.6%) isolates in a fish processing plant belonged to the group 1/2a–3a [13]. The present study showed that the majority of the isolates belonged to serotypes 1/2b (35.14%), 1/2a, and 1/2c (18.92%), which were the serotypes predominantly found in food. Serotype 4b was obtained mainly from patients with listeriosis and was not found in these isolates.

The antimicrobial resistance of *L. monocytogenes* is usually low (2–3%) [23, 26]. However, several studies have shown that up to 7.1% of strains resistant to antibiotics are not uncommon in fish processing plants [13]. In the present study, the commonly used antibiotics ceftazidime, ciprofloxacin, and lincomycin were generally ineffective against resistant *L. monocytogenes* isolates. This was probably because antibiotics were used in the breeding process. With the emergence of strains harboring antibiotic-resistant genes, such genes can be transferred between strains via plasmids. Multidrug resistance tests indicated that 90% of the isolates were resistant to more than two antibiotics, meaning that the antimicrobial resistance in *Listeria* was still low compared with that in the meat processing environment [7]. In recent years, a growing body of evidence suggests that the resistant bacteria produced in the processing environment may affect antibiotic resistance transfer in human pathogens through food products. Although many *L. monocytogenes* strains from humans are susceptible to antimicrobials, our results illustrated how new isolates could become resistant to commonly used antimicrobials.

In the present study, we first described multiple resistance genes *tetA*, *tetM*, *ermA*, *ermB*, *ermC*, and *aac(6′)-Ib* of *L. monocytogenes* isolated from the slaughter and processing environment. In general, *tetB* and *tetM* were frequently detected in mobile plasmids [27]. In our study, *tetA* and *tetM* were the major phenotypes, and these were significantly enhanced compared with those in previous studies, suggesting a potential connection of *tetA* and *tetM* with multidrug-resistant bacteria. The multidrug-resistant *L. monocytogenes* isolated from frozen food products harbors the multiple-resistant *ermB* and *tetS* genes [8]. Moreover, certain antibiotic-resistant genes such as *tetM* can be transferred among bacterial communities in various environments [28–30]. Horizontal gene transfer among humans and the environment is possible. The *cfr* gene was not identified in any of the 59 isolates, although this gene is commonly found in *staphylococcal* isolates from humans and animals [29]. Many reports on *cfr* genes come from China, mostly from animals. However, reports on the *cfr* gene in *Listeria* are few. Our analysis of resistant *Listeria* phenotypes and resistance genotypes found that the coincidence rate was inconsistent (Table 2), which might be due to the existence of multiple resistance mechanisms. In the present study, *Listeria* isolates were resistant to ampicillin (1.69%), erythromycin (49.15%), gentamicin (3.39%), and tetracycline (16.95%). The present findings partially correlated with those of Yadav et al. [31], who reported resistance to ampicillin, erythromycin, gentamicin, and tetracycline as 22.92, 16.67, 31.25, and 10.42%, respectively. Kumar et al. [32] reported that the multidrug-resistant *Listeria* isolated from meat and fish had sensitivity (66.66%) for ciprofloxacin. However, our study showed that the sensitivity of ciprofloxacin was 3.39%. Also, 91.38% strains of *Listeria* spp. resistant to three to six antibiotics were found. Therefore, it is of great concern that this expanding range of antibiotics now includes drugs used to treat human and animal listeriosis. The high number of multidrug-resistant strains of *Listeria* found in this study suggests that mobile genetic elements encoding resistance to a wide range of antibiotics in this genus have appeared and are spreading. The resistance mechanisms of bacteria are very complex. The location of resistance genes (on plasmids or chromosomes), genetic structure, expression level, interactions between different resistance genes, and formation of bacterial biofilms affect bacterial resistance to antibiotics. The resistance of bacteria to a drug may result from the combination of several resistance genes and resistance mechanisms [33]. Some studies have shown that *L. monocytogenes* can acquire resistance genes from the environment through plasmids and transposons, leading to the gradual increase in *L. monocytogenes* resistance [34]. Strains carrying the same antibiotic-resistant genotype may have different resistant phenotypes due to differences in the actual gene expression levels or antibiotic metabolism.

MLST plays an important role in analyzing the mode of contamination and transmission routes of *Listeria* [35]. Compared with European countries, the STs in the food processing environment mainly include ST1, ST9, ST87, ST5, ST7, ST37, ST570, and ST204. However, our study found that the major STs were ST5, ST9, and ST121. Almost all of ST9 and ST121 isolates were from the visceral area (C) and the meat cooling and refrigeration area (D). ST5 was isolated from all areas, indicating that ST5 played an important role in the entire processing environment. The C and D areas in the slaughtering processing environment contained large numbers of ST9 and ST121, indicating that ST9 and ST121 from RTE meat products might have originated from processing raw meat in the processing environment. In a study on 300 clinical, food, and environmental sources of isolates from 42 countries on 5 continents, CC9 was the fourth most common CC worldwide, ranking third in Europe after CC1, CC2, and CC3 [36]. In our study, among the seven housekeeping gene alleles, more than five identical alleles were present from a clonal complex. The main clonal complexes present in this study were CC5, CC9, and CC121. In the *L. monocytogenes* strains CC9 and CC121, premature stop codons leading to the truncation of the virulence gene *inlA* are often present. The *L. monocytogenes* STs are assigned to the latter cells and are considered to be more suitable for environmental conditions. Lineage II bacteria, including the most dominant worldwide strain CC121, are the main STs reported in human sporadic listeriosis. ST5, ST9, and ST121 included resistant isolates and resistance genes, suggesting that the monitoring of potentially pathogenic STs should be strengthened. ST5 has been associated with human listeriosis outbreaks, and ST9 is predominant in China [35, 37, 38], indicating that *L. monocytogenes* isolates in the slaughtering and processing environment share a common source with humans.

## Conclusions

In conclusion, the presence of serogroups 1/2a, 3a and 1/2b, 3b, as well as the resistance and pathogenic STs, was associated with human listeriosis. The findings of this study illustrated a potential public health risk in the slaughtering and processing environment. The greater resistance to antibiotics, particularly those commonly used to treat listeriosis, provides useful information for effectively treating *L. monocytogenes* infections. We found the three dominant STs in Jiangsu province, highlighting the need to fill out the MLST database by increasing the surveillance of *L. monocytogenes* worldwide.

## Materials and methods

### Sample collection and isolation of *listeria*

A total of 320 environmental swabs were collected from a pig slaughtering and processing environment in Jiangsu, China in 2019. The slaughtering and processing region can be divided into four areas: slaughter, carcass partition, visceral separation, and meat cooling and cryopreservation. Sampling included both food contact surfaces and non-food contact surfaces, including flooring, tables, walls, conveyor belts, trays, carts, and sinks [39]. A total of 320 environmental samples were collected at 2 times in 4 regions, including 80 from slaughter (flooring, carcass surface with pig hair, blood, sinks), 80 from carcass partition (carcass, cutting knife, conveyor) before their cleaning, 80 from visceral separation (cutting knife, tables, trays, walls), and 80 from meat cooling and cryopreservation (flooring, tables, trays, carts, belts, walls). About 100 cm^2^ of plane surfaces were swabbed two to five times using sterile cotton-tipped applicators moistened with 0.1% peptone water. The two to five swabs were pooled as one sample. The effluent was collected using sterile sampling bags. All of the samples were loaded in a refrigerated vehicle and transported to the lab within 24 h. *Listeria* was isolated according to the National Standard of China GB 4789.30–2016. For the detection of *Listeria* spp., 25 g of slaughterhouse samples were enriched in semi-concentrated Fraser broth (Merck, Germany) (primary selective broth) at 37 °C for 24 h, followed by transferring of the 0.1 mL of the initial base solution to 10 mL of Fraser broth (secondary selective broth) and incubation at 37 °C for 24 h. The enrichments were streaked onto Oxford agar (Merck, Germany) and Palcam agar (Merck, Germany) and incubated at 35 °C for 48 h. The plates were examined for *Listeria* colonies (black colonies with a black sunken center), and at least three suspected colonies were subcultured onto tryptone soy agar supplemented with 0.6% of yeast extract (Merck, Germany) and incubated at 37 °C for 24 h. All of the isolates were confirmed to possess the morphological characteristics of colonies and single bacterial cells after the Gram staining, catalase test, and motility test and using the API Listeria® (BioMérieux, Marcy I’Etoile-France) [in *Listeria* motility medium (Merck, Germany) after incubation at 25 °C for 2–5 days]. The serotyping of *L. monocytogenes* was carried out using the serum agglutination test according to the *Listeria* antisera of antigen 0 and flagellar antigen H (Denka Seiken Co. Ltd.).

### Antimicrobial susceptibility testing

Minimum inhibitory concentrations of *Listeria* isolates were determined using the microbroth dilution method recommended by the Clinical and Laboratory Standard Institute (CLSI, 2014). The following antimicrobial agents (Solarbio Ltd., China) were used in this study (range in μg/mL): gentamicin (GEN; 1–128), ampicillin (AMP; 2–128), ceftazidime (CAZ; 2–128), ciprofloxacin (CIP; 0.25–64), tetracycline (TET; 1–64), erythromycin (ERY; 0.25–16), lincomycin (LIN; 0.25–32), and vancomycin (VAN; 1–128). *Streptococcus pneumoniae* ATCC 49619 was selected as a quality control strain. Further, *tetA*, *tetM*, *tetS*, *ermA*, *ermB*, *ermC*, *aac*(*6′*)*-Ib*, *mecA*, *vanA*, *vanB*, and *cfr* were selected as specific resistance genes and were identified by PCR (Table 5).

**Table 5:** Primer used in this study for amplification of resistance genes of the *Listeria spp*

Category	Gene	Primer	Size (bp)	Accession number	Reference
Tetracycline	*tetA*	F:GCTACATCCTGCTTGCCTTC	220	NG_048154.1	[8, 28]
		R:CATAGATCGCCGTGAAGAGG			
	*tetM*	F:GTGGACAAAGGTACAACGAG	974	NC_013929.1	
		R:CGGTAAAGTTCGTCACACAC			
	*tetS*	F:CATAGACAAGCCGTTGACC	1050	NC_013929.1	
		R:ATGTTTTTGGAACGCCAGAG			
Aminoglycosides	*aac(6′)-Ib*	F:TTGCGATGCTCTATGAGTGGCTA	544	NZ_CP016990.1	[8]
		R:CTCGAATGCCTGGCGTGTTT			
Macrolides	*ermA*	F:AAGCGGTAAAACCCCTCGAG	651	MH_830363.1	
		R:TCA AAGCCTGTCGGATTGG			
	*ermB*	F:GAAAAGGTACTCAACCAAATA	639	NG_047798.1	
		R:CATTTGTTAAATTCATGGCAATGA			
	*ermC*	F:TCAAAACATAATATAGATAAA R:GCTAATATTGTTTAAATCGTCAAT	641	NG_047806.1	
ESBLs	*mecA*	F:TAGAAATGACTGAACGTCCG	154	NG_047937	[29]
		R:TTGCGATCAATGTTACCGTAG			
Vancomycin	*van A*	F:GGGAAA ACGACAATTGC	732	NC_011916.1	[8]
		R:GTACAA TGCGGCCGTTA			
	*van B*	F:TTGATGTGGCTTTCCCGGTT	544	NC_011916.1	
		R:ACCCGATTTCGTTCCTCGAC			
Multi-drugefflux pump gene	*cfr*	F:CGATTTGAGGATATGAAGGTTCT	416	NG_047631.1	[16]
		R:AAATTAGGATCCGTAAACGAAT			

### MLST

MLST based on seven housekeeping genes (*abcZ, bglA, cat, dapE, dat, ldh*, and *lhkA*) was performed by the method proposed by Wang et al. [35] The scheme and genotypic data are available at http://bigsdb.web.pasteur.fr/listeria/. Minimum spanning tree analysis was inferred using BioNumerics (Version 5.10, Applied Maths, Belgium).

### Virulence gene and invasion assays in vitro

Virulence genes *prfA*, *plcA*, *gyrB*, *plcB*, *inlA*, *hly*, and *sigB* of *L. monocytogenes* were identified by PCR as described previously [40, 41]. Primers and the size of each amplified product are listed in Table 6. The invasiveness of these isolates was measured using the human colorectal adenocarcinoma cell line Caco-2. In brief, Caco-2 cells (3.0 × 10^5^ cells per well) were cultured in 24-well plates in Dulbecco’s modified Eagle’s medium (DMEM) (Gibco; Invitrogen, CA, USA) containing 10% calf serum (Invitrogen) at 37 °C in an incubator supplemented with 5% carbon dioxide (CO_2_). Isolates of *L. monocytogenes* were grown in brain heart infusion broth under cultivation conditions of 30 °C for 18 h. Cell monolayers were infected with 1.0 × 10^7^ to 2.0 × 10^7^ *L. monocytogenes* cells/well for 30 min, followed by three washes with Dulbecco’s phosphate-buffered saline (DPBS). After incubating for 45 min, monolayers were overlaid with DMEM containing 100 μg/mL gentamycin to kill extracellular bacteria. After incubating for 90 min, the cells were washed three times with DPBS. Then, 1 mL of ice-cold distilled water was added, and viable intracellular bacteria were enumerated by plating appropriate dilutions of the cell lysate on BHI agar. At least three independent invasion assays were performed for each isolate. The invasion efficiency was calculated as the percentage of the inoculum recovered from the infected Caco-2 cells by the enumeration of intracellular bacteria [33, 39].

**Table 6:** Primer used in this study for amplification of virulence genes of *L. monocytogenes*

Gene	Primer	Size(bp)	Accession number	Reference
*prfA*	F:AGCGAGAACGGGACCATC	285	EU372057.1	[33]
	R:TTGACCGCAAATAGAGCC			
*plcA*	F:CCCAGAACTGACACGAGC	293		
	R:GCAGCATACTGACGAGG			
*gyrB*	F:AGACGCTATTGATGCCGATGA	91		
	R:GTATTGCGCGTTGTCTTCGA			
*plcB*	F:ATTAACCAAACCACTGGCTCA	502		[41]
	R:TTGATAAGCAGTCTGGACAAT			
*inlA*	F:ATAAGTGATATAAGCCCAG	606		
	R:TTTATCCGTACTGAAATTCC			
*hly*	F:GTTGCAAGCGCTTGGAGTGAA	420		
	R:ACGTATCCTCCAGAGTGATGG			
*sigB*	F:CCAAAAGTATCTCAACCTGAT	642		
	R:CATGCATTTGTGATATATCGA			

### Statistical analysis

Using SPSS 16.0 statistical software (SPSS Inc., IL, USA), a chi-square test was performed, and differences were considered significant at *P* values of < 0.05.

## Data Availability

Not applicable.
